# Nitric oxide-producing monocyte-myeloid suppressor cells expand and accumulate in the spleen and mesenteric lymph nodes of *Yersinia enterocolitica*-infected mice

**DOI:** 10.3389/fcimb.2024.1440514

**Published:** 2024-10-28

**Authors:** Marianela Leporati, María Silvia Di Genaro, Ricardo Javier Eliçabe

**Affiliations:** ^1^ División de Inmunología, Facultad de Química, Bioquímica y Farmacia, Universidad Nacional de San Luis, San Luis, Argentina; ^2^ Laboratorio de Inmunopatología, Instituto Multidisciplinario de Investigaciones Biológicas San Luis (IMIBIO-SL), Consejo Nacional de Investigaciones Científicas y Técnicas (CONICET), San Luis, Argentina

**Keywords:** Yersinia enterocolitica, Mo-MDSC, infection, nitric oxide, suppression

## Abstract

**Introduction:**

*Yersinia enterocolitica* (Ye) is a Gram-negative bacterium that causes gastrointestinal infections. The myeloid-derived suppressor cells (MDSCs) constitute a cellular population with the capacity of inducing the specific suppression of T cells. Although there is evidence supporting the role of MDSCs in controlling the immune responses in several bacterial infections, its role during Ye infection has not yet been reported. Therefore, the purpose of the present work was to analyze MDSCs after oral Ye infection.

**Methods:**

C57BL/6 wild-type mice were infected with Ye WAP-314 serotype O:8. The proliferation of splenocytes and mesenteric lymph nodes (MLN) cells was measured as well as the levels of cytokines and nitric oxide (NO) in culture supernatants. The frequency and subsets of MDSCs were analyzed in the intestinal mucosa and spleen by flow cytometry. Furthermore, monocytic-MDSCs (Mo-MDSCs) and polymorphonuclear-MDSCs (PMN-MDSCs) were purified from the spleen of infected mice and their suppressor activity was evaluated in co-cultures with purified T cells.

**Results:**

we observed a marked expansion of CD11b+Gr-1+ cells, a phenotype consistent with MDSCs, in the spleen and intestinal mucosa of Ye-infected mice. Interestingly, a robust proliferation of splenocytes and MLN cells was observed only when the MDSCs were depleted or the NO production was blocked. In addition, we determined that only Mo-MDSCs had the ability to suppress T-cell proliferation.

**Conclusion:**

Our results highlight a mechanism by which Ye may induce suppression of the immune responses. We suggest that NO-producing Mo-MDSCs expand and accumulate in MLN and spleen of Ye-infected mice. These cells can then suppress the T-cell function without interfering with the anti-bacterial effector response. Instead, these immature myeloid cells may perform an important function in regulating the inflammatory response and protecting affected tissues.

## 1 Introduction

*Yersinia enterocolitica* (Ye) is a predominant extracellular Gram-negative bacterium that causes gastrointestinal infections, mesenteric lymphadenitis and can lead to systemic infection (Fàbrega and Vila, 2012). Usually, the infection by Ye occurs after consumption of contaminated food or water (Fredriksson-Ahomaa et al., 2006; Chlebicz and Śliżewska, 2018). After infection, Ye invades M cells in the Peyer’s patches (PP), especially those of the distal small intestine (terminal ileum), then they can invade mesenteric lymph nodes (MLN) and eventually reach the spleen, liver and lung. During the infection, innate and adaptive immune responses are critical for controlling Ye dissemination (Autenrieth et al., 1993; Conlan, 1997; Autenrieth et al., 2010, 2012). Nevertheless, it has been proven that Ye can evade the immune system to favor its survival in the host tissues (Fàbrega and Vila, 2012). In inflammatory conditions, such as acute or chronic infections, there is an increased demand of myeloid cells to renew or restore the peripheral populations that are consumed. This process, known as emergency myelopoiesis, mainly occurs in the bone marrow (BM). Moreover, to respond to this hematopoietic emergency, hematopoiesis can occur in organs outside of the BM, including the spleen, considered as a major site for extramedullary hematopoiesis (Oda et al., 2018). During inflammatory conditions, progenitors and immature myeloid cells (IMCs) migrate from BM to the peripheral tissues, where they mature into monocytes, macrophages, dendritic cells or polymorphonuclear granulocytes (Millrud et al., 2017). However, in certain conditions, cytokines (GM-CSF, M-CSF, G-CSF, IL-6, IL-10, IFN-γ, IL-1β) and pro-inflammatory factors (PAMPs, polyunsaturated fatty acids, DAMPs, alarmins such as S100A9 and A8, PGE2) produce the accumulation of IMCs. The microenvironment of the inflamed area blocks the terminal differentiation of these immature precursors and induces their pathological activation. These immature precursors normally exhibit immunosuppressive functions and are therefore known as myeloid-derived suppressor cells (MDSCs) (Gabrilovich and Nagaraj, 2009; Millrud et al., 2017; Veglia et al., 2018). MDSCs are defined as a heterogeneous cellular population with distinct morphologic and phenotypical characteristics that exhibit the capacity to induce the specific suppression of T-cells (Gabrilovich and Nagaraj, 2009). Although they were first described in cancer, they have lately gained remarkable relevance in different infectious diseases. Thus, the presence of MDSCs has been described in infections caused by viruses, bacteria, fungi and parasites (Ost et al., 2016). In mice, MDSCs are defined as cells expressing both Gr-1 and CD11b markers. Based on their phenotypic and morphological features, the MDSCs can be classified into two major subsets: polymorphonuclear-MDSCs (PMN-MDSCs) and monocytic-MDSCs (Mo-MDSCs). PMN-MDSCs can be defined as CD11b+Ly6G+Ly6Clow cells with granulocyte-like morphology and elevated arginase-1 (Arg-1) expression. In contrast, Mo-MDSCs can be defined as CD11b+Ly6G−Ly6Chi cells that have similar morphology as monocytes and preferably express the enzyme inducible nitric oxide synthetase (iNOS) (Bronte et al., 2016). As previously mentioned, the main feature of MDSCs is their strong ability to induce immunosuppression. However, PMN-MDSCs and Mo-MDSCs use different mechanisms to suppress immune responses. The most prominent factors implicated in MDSCs suppressive activity are mainly linked to the metabolism of L-arginine (L-Arg), including nitric oxide (NO) production and L-Arg depletion. In addition, the up-regulation of reactive oxygen species (ROS) and peroxynitrite, the Treg induction and the production of immunoregulatory cytokines are other ways by which MDSCs carry out their suppressive activity (Gabrilovich and Nagaraj, 2009; Veglia et al., 2018). Although there is evidence supporting the role of MDSCs in controlling the immune responses in several bacterial infections (Ost et al., 2016; Goldmann and Medina, 2023; Zhang et al., 2023), their suppressive activities during Ye infection have not yet been reported. Therefore, the purpose of this work was to analyze MDSCs after oral Ye infection. Here we observed a marked expansion of CD11b+Gr-1+ cells, a phenotype consistent with MDSCs, in the spleen and intestinal mucosa. Interestingly, a robust proliferation of splenocytes and MLN cells was observed only when the MDSCs were depleted or the NO production was blocked. In addition, we determined that only Mo-MDSCs had the ability to suppress T-cell proliferation. Our results contribute to the knowledge of MDSCs in bacterial infection settings. These findings can be applied to design treatments that have MDSCs as targets without altering the protective immune response, limiting the immunopathology and sequelae of these infections.

## 2 Materials and methods

### 2.1 Mice

C57BL/6 wild-type mice were purchased from the Animal Facilities of the National University of La Plata (La Plata, Argentina). Breeding colonies were established at the Animal Facility of the National University of San Luis (San Luis, Argentina). Mice were kept under specific pathogen–free conditions in a cabinet (Ehret, Emmendingen, Germany) and provided with sterile food and water ad libitum. Female mice of 8-12 weeks-old were used for all the experiments. All animal procedures were performed according to the rules and standards for the use of laboratory animals of the U.S. National Institutes of Health (NIH). Animal experiments were approved by the Institutional Committee of Care and Use of Animals (CICUA) of the Faculty of Chemistry, Biochemistry and Pharmacy at the National University of San Luis (San Luis, Argentina) (protocols numbers B-256/17, B-256/19).

### 2.2 Bacterial strains and infection

Ye serotype O:8 WAP-314 pYV, kindly provided by Dr. Autenrieth (Tuebingen, Germany) was used in this study. Bacteria were cultured as previously described (Di Genaro et al., 2003). Bacteria were grown in Luria broth at 27°C, harvested during the log phase, and frozen in 1 ml aliquots at -80°C. Prior to each experiment, an aliquot was thawed, washed, and resuspended in sterile saline. Mice were infected orogastrically, with 1-5×10^8^ yersiniae in 200 μl saline by gavage. The actual bacteria number administrated was controlled for each experiment by plating serial dilutions of the inoculated suspension on Mueller–Hinton agar by duplicate and counting the colony-forming units (CFUs) after incubation at 27°C for 48 h. For the determination of the bacterial burden after infection, PP, MLN and spleen were aseptically obtained and homogenates were prepared in Hanks’ balanced salt solution (HBSS). Serial dilutions of these homogenates were plated on Irgasan-MacConkey agar plates for PP or Mueller-Hinton (MH) plates for MLN and spleen samples. Plates were incubated for 48 h at 27°C and CFUs were determined.

### 2.3 Cell preparation and flow cytometry

A total of 6 and 8 PP were collected from the entire small intestine. Furthermore, between 4 and 5 MLN were collected from the intestinal mesentery. Single-cell suspensions of PP, MLN and spleens were prepared for mechanical disintegration and homogenates were passed through a 70 μm cell strainer. The spleen erythrocytes were lysed with an ammonium-chloride-potassium (ACK) lysing buffer. For the identification of MDSCs, cells were stained in PBS containing 0.05% sodium azide and 2.5% fetal bovine serum (FBS) with anti-mouse CD11b-FITC (BD Pharmingen, clone M1/70) and GR-1-PE (BD Pharmingen, clone RB6-8C5) for 30 min in ice. For the identification of PMN-MDSC and Mo-MDSC, cells were stained with anti-mouse CD11b-FITC, Ly-6G-PE (Biolegend, clone 1A8) and Ly-6C-PerCP-Cy5.5 (Biolegend, clone HK1.4) as described above. To analyze Treg cells, the cells were stained using a Mouse Regulatory T Cell Staining Kit (eBioscience) according to the manufacturer’s instructions. In order to standardize the results, the numbers of cells were expressed per PP or MLN. Data were acquired using a BD FACSCalibur flow cytometer (BD Biosciences, San Diego, CA, USA) and analyzed with FlowJo software version 7.6 (Tree Star, Ashland, OR).

### 2.4 *In vitro* culture

Isolated splenocytes and MLN cells were resuspended in a complete tissue culture medium (RPMI supplemented with 10% FBS, 2 μmol L-glutamine, 100 U/ml penicillin, and 100 mg/ml streptomycin, 1 mM Pyruvate and 50 μmol/ml 2-ME), cultured in 96-well plates at 37°C in an atmosphere of 5% CO_2_ (1 x10^6^ viable cell/well) and stimulated with 1x10^8^ bacteria/ml of heat-killed *Yersinia* (HKY) or 5 μg/ml of Concanavalin A (ConA) for 3 days. Culture supernatants were obtained for nitrite quantification or stored at -20°C until cytokine determination. Each sample was analyzed in duplicate, and the resulting data were expressed as the mean of the duplicates.

### 2.5 Nitric oxide and cytokine measurement

Nitric oxide was measured in culture supernatants as nitrite accumulation using the Griess reaction (Griess, 1858). Fifty microliters of supernatants were put into a 96-well plate. Simultaneously, a standard curve of NaNO_2_ in the medium was prepared. An aqueous solution of 5.5 nM of naphtylethylendiamine and 1.5% sulfanilamide in 1 N chloride acid were mixed 1:1 and 100 μl of this solution was added to 50 μl of the samples and NaNO_2_ standards. The color reaction was measured after 10 min on a plate reader (Epoch, BioTek Instrument, Winooski, Vermont, US) at 550 nm and nitrite concentrations were calculated from the NaNO_2_ standard curve.

### 2.6 Isolations of CD4+ T cells and MDSC

Splenic CD4+ T cells were purified using anti-mouse CD4 magnetic particles (BD IMag 551539) following the manufacturer’s instructions. The isolation of subpopulations of MDSCs from spleen cell suspensions was performed using the commercial Mouse Myeloid-Derived suppressor Cell Isolation kit (Miltenyi Biotec, Bergisch Gladbach, Germany) following the manufacturer’s instructions. The purity of the isolated populations was controlled by flow cytometry.

### 2.7 Proliferation assays in splenocytes and MLN cells

To assess cell proliferation, splenocytes and MLN cells were prepared and stimulated with HKY or ConA for 3 days as described above. Then, the plates were incubated with 0.2 mg/ml MTT (Sigma) for an additional period of 4 h. To dissolve the insoluble purple formazan product, a solution of DMSO and isopropyl alcohol (1:1 ratio) was added. The colored solution absorbance was quantified at 570 nm with a plate reader. Alternatively, cell proliferation was analyzed by flow cytometry. For that, splenocytes or MLN cells were labeled with carboxyfluoresceinsuccinimidyl ester (CFSE) dye, plated in 96-well plates and stimulated with HKY or ConA for 6 days and analyzed by flow cytometry.

### 2.8 T cell proliferation and MDSC inhibition assay

To assess suppression activity of Mo-MDSCs or PMN-MDSCs, purified CD4+ T cells labeled with CFSE were co-cultured with purified Mo-MDSCs or PMN-MDSCs from Ye infected-mice using a 1:1 ratio (CD4+ T cells/MDSCs) and stimulated with ConA. After 6 days, cells were stained with anti-mouse CD4-PE (BD Pharmigen, clone) and analyzed by flow cytometry.

### 2.9 *In vivo* depletion of MDSC

Mice were orogastrically infected with Ye. On days 1 and 3 after infection, the mice were treated with 120 mg/Kg of Gemcitabine (GEM) via intraperitoneal (I.P.) diluted in saline. Control mice received saline alone. At 5 days p.i, spleen and MLN cells were isolated to perform the experiments. The MDSCs depletion was controlled by flow cytometry.

### 2.10 *In vitro* treatments

To assess the suppressive mechanisms of MDSCs during Ye infection, the cell cultures were supplemented with iNOS inhibitor aminoguanidine (AG, 0.5 mM; Sigma-Aldrich) or L-Arg (2.5 mM; Biopack) and stimulated with ConA or HKY. Cell proliferation, nitrite and IFN-γ were evaluated after 3 days.

### 2.11 Statistics

Results were expressed as mean ± SEM of the duplicate samples of two or three independent experiments. They were analyzed by Two-tailed Student t test or One-way ANOVA with *post-hoc* Tukey test correspondingly, using GraphPad Prism 5.0 software (GraphPad Software, La Jolla, CA, USA). Differences were considered significant when the p-value was less than 0.05.

## 3 Results

### 3.1 Ye regulates the proliferative response of splenocytes

**Figure 1:**
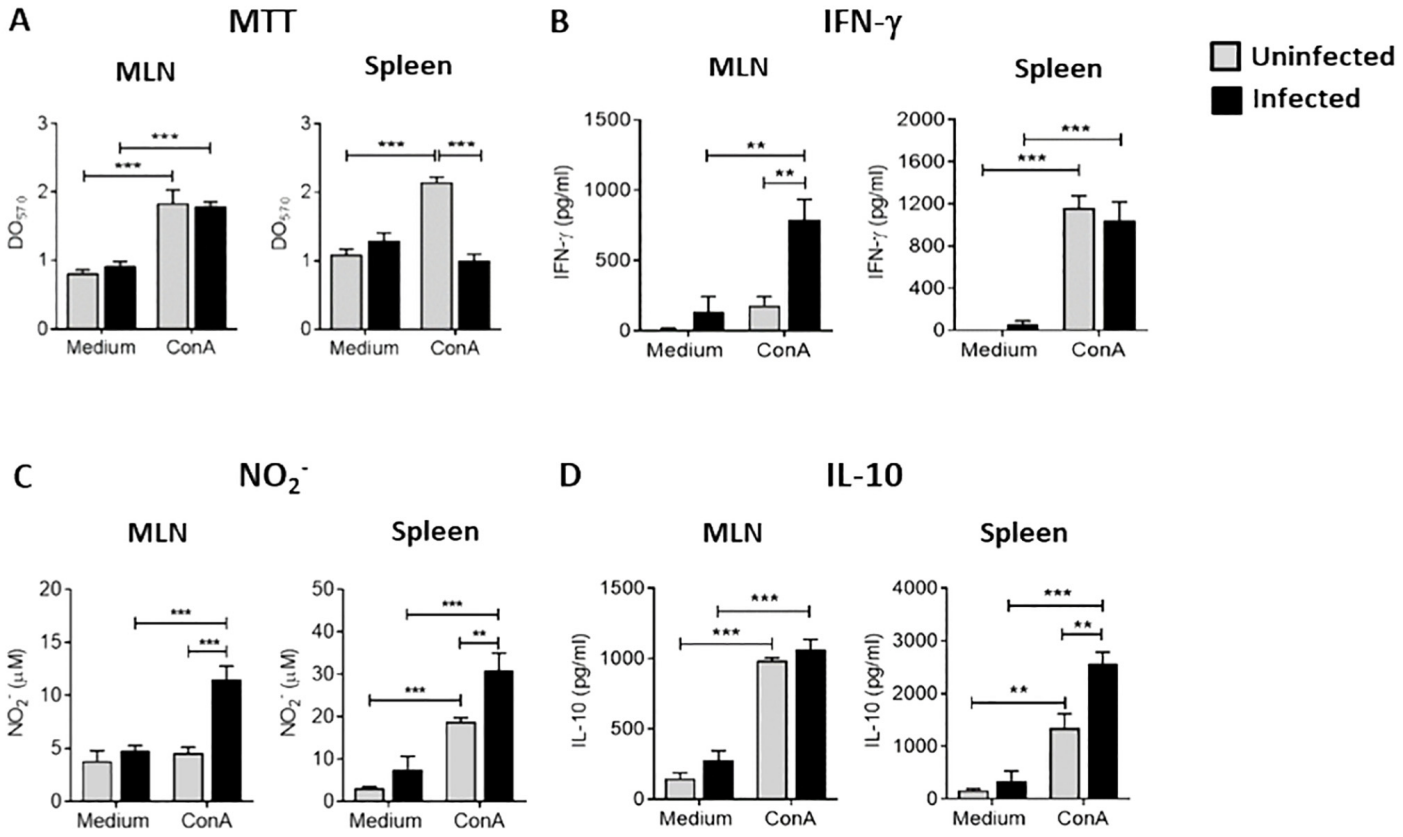
Suppression of T cell proliferation in the spleen of Ye-infected mice. Mice were intragastrically infected with Ye. At day 5 p.i, MLN and spleen were collected and cell suspension was prepared and cultured in 96 well plates (1 x10^6^ viable cells/well). The T-cell proliferation was triggered using 5 μg/ml of ConA for 72 h **(A)** Proliferation in MLN and spleen of infected and uninfected mice determined by MTT assay. Levels of IFN-γ **(B)**, NO **(C)** and IL-10 **(D)** in culture supernatants of MLN cell and splenocytes. Each group included from 5 to 8 mice. Results are expressed as means ± SEM of two independent experiments. Data were analyzed using a one-way ANOVA, ** *p* < 0.005; *** *p* < 0.001. OD, optical density.

We first examined whether Ye infection could modulate the proliferation of splenocytes and MLN cells. For this purpose, splenocytes and MLN cells of infected and non-infected mice were stimulated *in vitro* with ConA for 72 h and cell proliferation was evaluated by MTT assay. As expected, the proliferation of MLN cells of infected and non-infected mice was significantly major after stimulation compared with non-stimulated cells. The same effect was observed in splenocytes from uninfected mice. Strikingly, splenocytes of infected mice showed similar proliferation levels to unstimulated cells and significantly lower than those of splenocytes from uninfected mice (**Figure 1A**). Further, we also assessed IFN-γ, NO, and IL-10 levels in culture supernatants of MLN cells and splenocytes stimulated with ConA. We observed a significant increase in the production of IFN-γ and NO in the MLN cultures of infected mice compared to those uninfected. Nevertheless, we could not find significant differences in IL-10 levels. In contrast, splenocytes from infected mice secreted similar levels of IFN-γ to those from uninfected mice. However, they produced higher amounts of NO and IL-10 (**Figures 1B–D**). This result suggests that a classical antibacterial response takes place in the intestinal mucosa during Ye infection, while the effector response seems to be down-regulated in the spleen.

### 3.2 Ye infection induces MDSCs accumulation in intestinal mucosa and spleen

**Figure 2:**
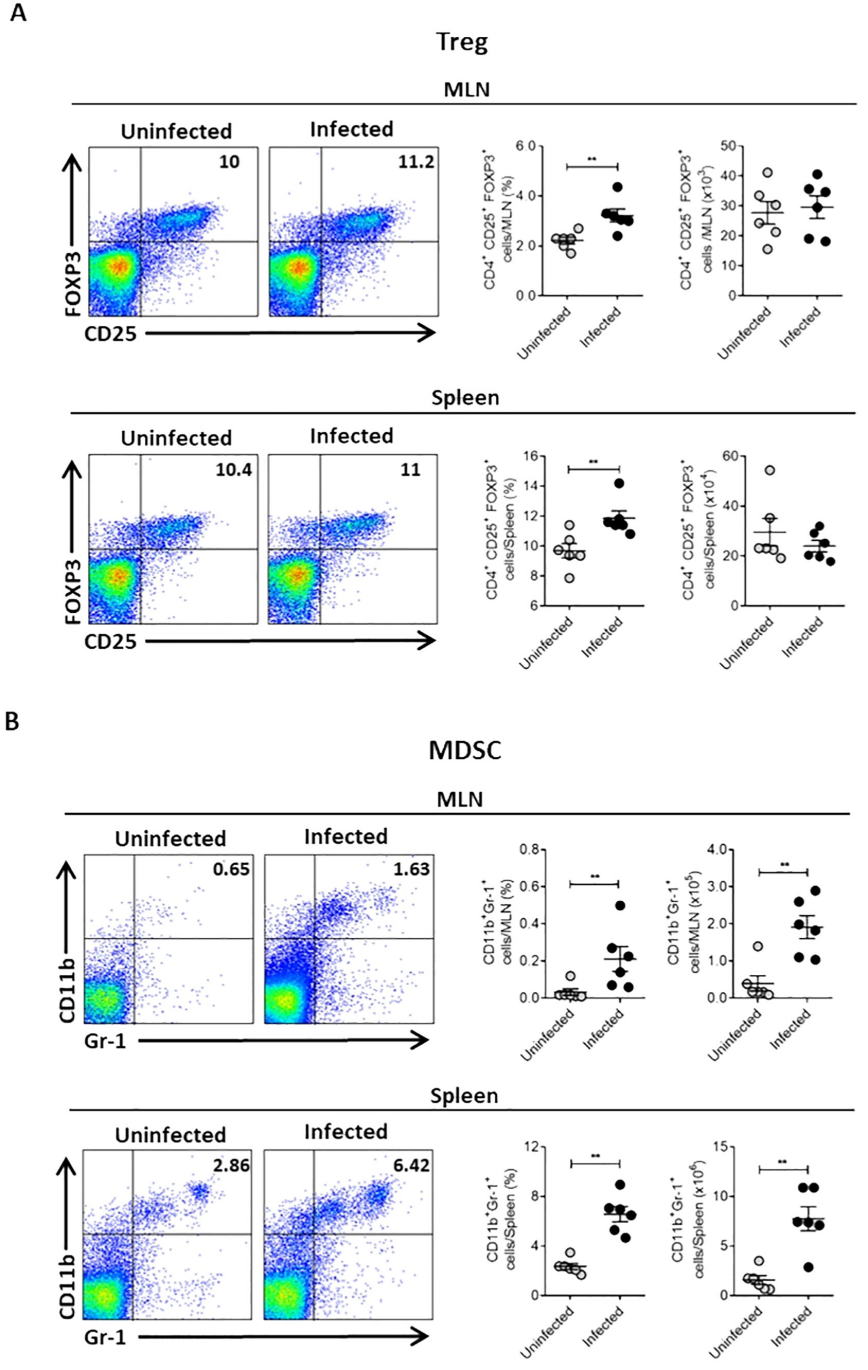
Oral Ye infection induces accumulation of Treg and MDSCs in intestinal mucosa and spleen. Mice were intragastrically infected with Ye. On day 5 p.i, mice were sacrificed and their MLN and spleen cells were stained to analyze MDSCs and Treg cells by flow cytometry. **(A)** Representative flow cytometry plots and dot plot graphics showing the percentage and absolute number of Treg cells in MLN (upper panel) and the spleen (lower panel) of uninfected or infected mice. **(B)** Flow cytometry plots and dot plot graphics showing the percentage and absolute number of CD11b+GR-1+ cells in MLN (upper panel) and the spleen (lower panel) of uninfected or infected mice. The MDSCs were identified as CD11b+Gr-1+ cells and the Treg as CD4+CD25+FOXP3+ cells. Each symbol represents a mouse (6 mice per group). Results are expressed as means ± SEM of two independent experiments. Differences between uninfected and infected mice were analyzed by t-test. ** *p* < 0.01.

**Figure 3:**
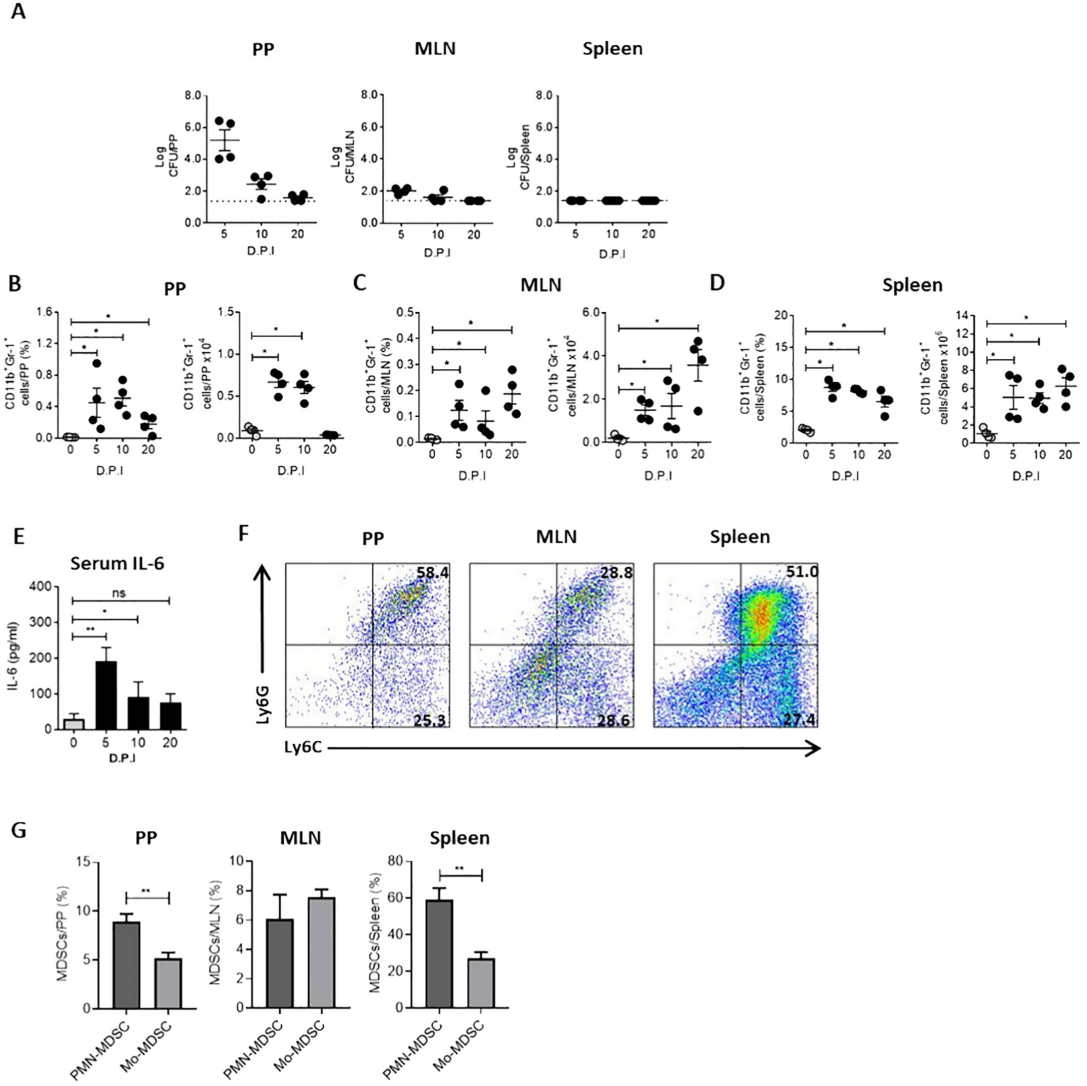
MDSCs accumulate and persist in the intestinal mucosa and spleen of Ye-infected mice. Mice were infected with Ye, on days 5, 10 and 20 p.i were killed and MDSCs presence was analyzed in PP, MLN and spleen. **(A)** Bacterial load in PP, MLN and spleen of Ye infected mice at different days p.i (D.P.I). The dotted line represents the detection limit (log CFU = 1.4). **(B)** Percentage and absolute number of MDSCs at different D.P.I in PP, MLN **(C)** and the spleen **(D)**. The MDSCs were identified as CD11b+Gr-1+ cells. Uninfected mice were used as controls. **(E)** IL-6 levels in sera collected at indicated D.P.I. **(F)** Flow cytometry plots showing phenotypic characterization of MDSCs subsets in PP, MLN and the spleen at day 5 p.i. Cells were gated on the CD11b+ population and analyzed for the expression of Ly6G and Ly6C. **(G)** Percentage of PMN-MDSC and Mo-MDSC in PP, MLN and the spleen at day 5 p.i. Each group included from 4 to 5 mice, A-D, each symbol represents a mouse. Results are expressed as means ± SEM of two independent experiments. Differences between uninfected and infected mice were analyzed by a t-test. * *p* < 0.05; ** *p* < 0.01; ns, not significant.

Several mechanisms can suppress the immune response during infection. Together with the suppression observed in the spleen of Ye-infected mice, we found a slight increase in Treg cells percentage in the spleen and MLN of infected mice (**Figure 2A**). This result indicates a minor role of Treg cells in the T cell suppression in the spleen of infected mice. However, we noted significant increases in the percentage and number of CD11b+Gr-1+ cells, a phenotype that characterizes MDSCs, in the spleen and MLN of infected mice (**Figure 2B**). This finding suggests that these cells could play a central role in the suppressive response observed during Ye infection. Moreover, we examined the bacterial load in PP, MLN and spleen at different days p.i. and compared these with the MDSCs presence in the same organs to confirm that Ye induce the expansion and accumulation of MDSCs in the intestinal mucosa and spleen. Our findings indicated that the infection resulted in the onset of inflammation, accompanied by an observable increase in the size of the PP, MLN and spleen. In addition, we observed a high bacterial load in the PP of infected mice at days 5 and 10 p.i., while the presence of bacteria was lower in MLN. At day 20 p.i., no bacteria were detected in PP and MLN, suggesting that Ye may have been eradicated from the intestinal mucosa at this time. There was no evidence of the presence of Ye in the spleen on any of the days tested (**Figure 3A**). Coinciding with the presence of Ye in the intestinal mucosa, the percentage and number of MDSCs in PP and MLN increased significantly at days 5 and 10 p.i. The percentage and number of MDSCs showed a marked decrease in PP at day 20 p.i., which was in line with the absence of bacteria in this organ (**Figure 3B**). Interestingly, although no bacteria were detected in the MLN at day 20 p.i., a marked increase of MDSCs was observed in this organ (**Figure 3C**). Even though Ye was not detected in the spleen of infected mice on any of the different days tested, we observed a significant and sustained increase in the percentage and number of MDSCs in this organ (**Figure 3D**). Since it has been reported that IL-6 is a crucial cytokine that regulates MDSCs accumulation and activation (Weber et al., 2021), we determined serum IL-6 in the mice. We found that the MDSCs accumulation in the intestinal mucosa and spleen of Ye-infected mice was accompanied by elevated levels of serum IL-6 at days 5 and 10 p.i. (**Figure 3E**). When the infiltrated MDSCs of PP, MLN, and spleen were classified as granulocytic or monocytic based on Ly6G or Ly6C expression, the analysis revealed a significant predominance of cells with a PMN-MDSC phenotype (CD11b+Ly6ClowLy6G+) in the PP and spleen, while in the MLN there was an equal distribution of PMN-MDSC and Mo-MDSC (CD11b+Ly6C+Ly6G-) (**Figures 3F, G**). These results indicate that Ye oral infection induces mobilization and accumulation of MDSCs in mucosal and systemic organs.

### 3.3 MDSCs are responsible for the immune response suppression in MLN and the spleen of Ye-infected mice

**Figure 4:**
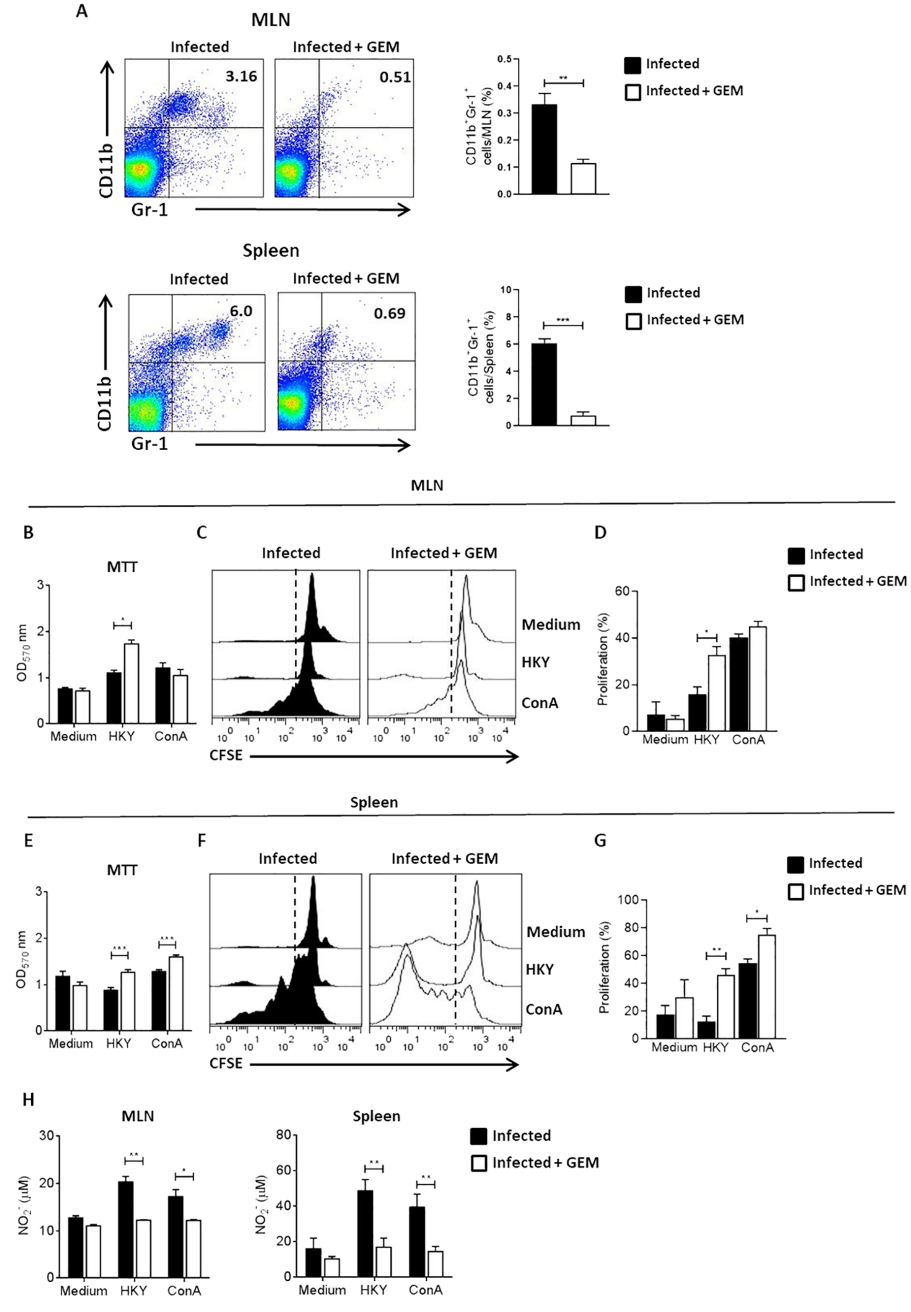
MDSCs suppress the proliferation of splenocytes and MLN leucocytes during Ye infection. On days 1 and 3 after infection, the mice were treated with 120 mg/kg of GEM to deplete MDSCs. On day 5 p.i, the mice were killed and leucocytes from MLN and splenocytes were cultured in the presence of 2 µg/ml ConA or 1x10^8^/ml HKY. After 72 h, cell proliferation was determined by MTT assay. Alternatively, the cells were stained with CFSE and the proliferation was analyzed by flow cytometry after 6 days of stimulation with ConA or HKY. **(A)** Dot plots show depletion of MDSCs in MLN and spleen of mice treated with GEM after 5 days compared to mice without further treatment. Plots show quantification of depletion results as assessed by flow cytometry. **(B)** Proliferation of MLN leucocytes obtained from non-depleted and MDSC-depleted mice were analyzed by MTT assay. **(C)** Representative histograms of CFSE dilution in MLN cells. **(D)** Quantification of each condition of MLN cells are shown. **(E)** Proliferation of splenocytes coming from non-depleted and MDSC-depleted mice analyzed by MTT assay. **(F)** Representative histograms of CFSE dilution in splenocytes. **(G)** Quantification of each condition of splenocytes are shown. The numbers in the histogram plots represent the percentage of proliferating cells. **(H)** NO levels in supernatants of ConA or HKY-stimulated MLN cells and spleen cells from non-depleted and MDSC-depleted mice. Each group included from 5 to 8 mice. Results are expressed as means ± SEM of at least two independent experiments. Data were analyzed using a one-way ANOVA. * *p* < 0.05; ** *p* < 0.01; *** *p* < 0.001;, GEM, gemcitabine; HKY, heat-killed *Yersinia*.

To determine whether MDSCs induced by Ye infection exert immunosuppression, we determined the cellular proliferation in the cell culture of MLN cells and splenocytes obtained from infected mice previously depleted of MDSCs. For this purpose, mice were infected and treated with GEM and MLN cells and splenocytes were stimulated with HKY or ConA. The proliferative activity was evaluated by MTT assay and confirmed by flow cytometry with the CFSE assay. First, to evaluate whether targeting GEM can effectively deplete MDSCs *in vivo*, we examined the frequency of MDSCs in infected mice that received two doses of GEM (120 mg/Kg). Five days after administration of the first dose of GEM, a significant depletion of MDSC was observed in the MLN and spleen (**Figure 4A**). Our results showed that the proliferation of MLN cells of MDSCs-depleted mice was significantly higher in comparison to non-depleted mice only after stimulation with the specific antigen (**Figure 4B–D**). Similarly, splenocytes coming from MDSCs-depleted-mice showed higher proliferation with both specific and polyclonal stimulus, compared to those of non-depleted mice (**Figure 4E–G**). In agreement with these results, a significant reduction in NO levels was detected in the culture supernatants of MLN cells and splenocytes from MDSCs-depleted-mice after specific antigen or ConA stimulation (**Figure 4H**). These data suggest that MDSCs expanded during Ye infection, accumulated in the spleen and MLN and exerted suppressive activity.

### 3.4 Mo-MDSCs but not PMN-MDSCs suppress CD4+ T-cell proliferation during Ye infection through an iNOS-dependent mechanism and NO production

**Figure 5:**
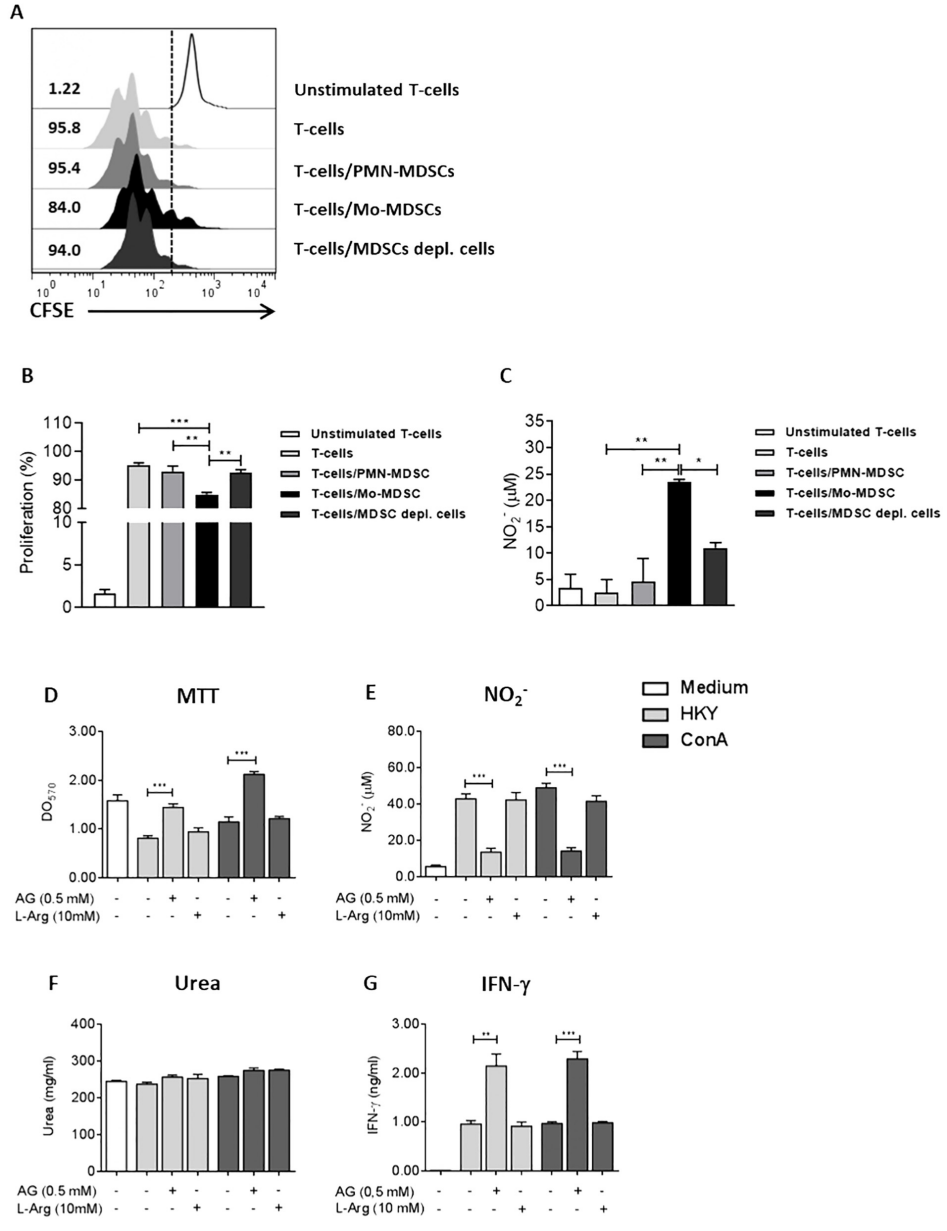
Mo-MDSCs purified from the spleens of Ye-infected mice inhibit T-cell proliferation through a NO-dependent mechanism. **(A)** At day 5 p.i, Mo-MDSCs and PMN-MDSCs were purified from the spleen of Ye-infected mice and they were co-cultured during 5 days with CFSE-labeled T-cells in the presence of 2 µg/ml of ConA. The ratio of MDSCs subpopulations and T cells was 1:1. ConA-stimulated T-cells, non-stimulated T-cells or T-cells co-cultured with the MDSCs-depleted fraction were used as control. The histograms show the proliferating CD4+ T-cells cultured in different conditions. **(B)** Quantitative analysis of CD4+ T-cells proliferation. **(C)** Nitrite levels in the supernatants of the co-cultures of T-cells and MDSCs subpopulation. **(D)** Proliferation of splenocytes of infected mice in the presence of iNOS inhibitor AG or amino acid L-Arg. At day 5 p.i, splenocytes were stimulated with 2 µg/ml of ConA or 1x10^8^/ml of HKY in the presence or absence of 0,5 mM AG or 10 mM L-Arg. After 72 h, the proliferation was evaluated by MTT assay. Nitrite **(E)**, urea **(F)** and IFN-γ levels **(G)** were measured in the supernatants of splenocytes stimulated with ConA or HKY in the presence or absence of AG or L-Arg. Each group included from 5 to 8 mice. Results are expressed as means ± SEM of three independent experiments. Data were analyzed using a one-way ANOVA. * *p* < 0.05; ** *p* < 0.01; *** *p* < 0.001. HKY, heat-killed Yersinia.

It has been demonstrated that Mo-MDSCs and PMN-MDSCs can inhibit T-cell function in several infections (Medina and Hartl, 2018). To determine whether both subpopulations are able to suppress T-cell function during Ye-infection, we isolated Mo-MDSCs and PMN-MDSCs from the spleen of infected mice. They were co-cultured with naïve CD4+ T-cells, stimulated with ConA and stained with CFSE. The proliferation of T-cells was examined in gated CD4+ cells by flow cytometry. We observed that the proliferation of stimulated T-cells co-cultured with Mo-MDSCs was suppressed, compared to those T-cells co-cultured with PMN-MDSCs, MDSC-depleted splenocytes or T-cells stimulated with ConA (**Figure 5A, B**). Furthermore, the NO levels in the supernatant of T cells co-cultured with Mo-MDSCs were significantly higher than those in the T-cells co-cultured with PMN-MDSCs or with MDSCs-depleted splenocytes (**Figure 5C**). In turn, the ability of MDSCs to inhibit T-cell responses has been linked to their increased L-Arg metabolism via the Arg-1 and iNOS pathways (Raber et al., 2012; Calderon et al., 2023). To confirm the pathway behind the MDSCs suppressive activity during Ye infection, splenocytes from infected mice were stimulated with HKY or ConA in the presence of AG to inhibit NO production. Furthermore, another group of cells was supplemented with L-Arg to compensate for the decrease of this amino acid caused by Arg-1 activity. We observed a robust proliferation in splenocytes treated with AG and stimulated with both HKY and ConA, compared to those cultured in the absence of this inhibitor. In contrast, the response was suppressed in cultures supplemented with L-Arg (**Figure 5D**). In line with this result, NO levels were significantly reduced in the supernatants of the cells stimulated and treated with AG. We also observed that the cells supplemented with L-Arg increased the production of NO (**Figure 5E**). Furthermore, no differences were observed in the urea levels, a product of L-Arg metabolism by Arg-1, when comparing cells supplemented with L-Arg with those that received only the stimulus, indicating that the L-Arg supplementation contributes to feeding the iNOS pathway (**Figure 5F**). In addition, the increased proliferative activity and reduced NO levels in splenocytes treated with AG and stimulated with HKY or ConA also showed increased IFN-γ levels in the supernatant, compared to those that only received the stimulus (**Figure 5G**). Together, these findings indicate that NO production, but not Arg-1, contributes to the suppression of T-cell proliferation by Mo-MDSCs from Ye-infected mice.

## 4 Discussion

MDSCs have been described in several infectious diseases such as *Salmonella enterica, Staphylococcus aureus, Pseudomonas aeruginosa, Klebsiella pneumoniae* and *Mycobacterium tuberculosis*. However, the role of these cells is still unknown or poorly understood in many infections (Peñaloza et al., 2019). Our study provides the first evidence that during Ye infection, MDSCs expand and suppress T-cell responses. In the last years, MDSCs have emerged as key players in balancing the inflammatory response and pathogenesis during an infection. Some pathogens can exploit the suppressive activities of these cells to favor pathogen persistence and chronic infections (Ost et al., 2016). We demonstrated that large amounts of MDSCs accumulate and persist in the spleen and MLN of mice infected with Ye. Moreover, we observed a strong suppressive effect on the immune response in the spleen of Ye-infected mice, which was associated with the presence of NO and IL-10. This immunosuppressive effect of NO on splenocytes of infected mice has also been demonstrated in *Salmonella* infections (Eisenstein et al., 1994). In addition, IL-10 is a crucial factor in enhancing the immunosuppressive network (Salminen, 2021). Although it has not yet been proved whether NO and IL-10 can act synergistically to suppress the immune response, there is evidence suggesting that other reactive species produced by myeloid cells, such as ROS, can boost the regulatory effects of IL-10 in a murine model of lung inflammation triggered by endotoxin (Deng et al., 2012). This suppressive effect observed in the spleen of infected mice was accompanied by a significant influx of MDSCs, as well as a lesser increase in Treg cells. Based on this observation, we cannot rule out that MDSCs infiltrate the spleen during infection and promote the differentiation of Treg cells as a source of IL-10, as has been previously reported (Serafini et al., 2008; Zoso et al., 2014). We found a classic protective response in the intestinal mucosa linked to the production of NO and IFN-γ. It is well established that the immune response to Ye infection is strongly associated with the Th1-type response, which is linked to the production of IFN-γ and NO (Bohn and Autenrieth, 1996; Tumitan et al., 2007). Even though the lymph nodes associated with the intestinal mucosa also showed a marked influx of MDSCs and Treg cells, no suppression of the immune response was observed. The analysis of MDSCs infiltration in the intestinal mucosa and spleen revealed that the accumulation of these cells increases with bacterial presence in PP, and decreases when the mouse overcomes the infection. In contrast, the behavior observed in the MLN and spleen was different. Although the bacterial load was low or negative in these organs, the infiltrating MDSCs increased and remained elevated even after the mice had overcome the infection. Since MDSCs persisted in MLN and spleen when bacterial counts are negative, we cannot exclude the possibility that low levels of non-culturable bacteria or bacterial antigens are present in the MLN and spleen of infected mice at those times. This could explain to some extent the persistence of MDSCs in these organs in the absence of bacteria. This would be supported by the fact that the expansion and accumulation of MDSCs is driven by chronic inflammation, a process that is fundamentally associated with sustained antigenic stimulation (Meyer et al., 2011). Furthermore, in mice, the spleen can act as an extramedullary myelopoiesis organ where MDSCs can expand (Millrud et al., 2017). The LPS has been demonstrated to induce the accumulation of MDSCs at extramedullary sites, especially the spleen (Massberg et al., 2007; Skabytska et al., 2014). Additionally, the rise in MDSCs migration to the intestinal mucosa and the spleen of infected mice was accompanied by an increase in IL-6 levels during the active infectious phase. This finding is supported by the evidence that IL-6 plays a critical role in the accumulation of MDSCs and the development of their immunosuppressive properties (Condamine and Gabrilovich, 2011; Weber et al., 2020, 2021).

Furthermore, it was observed that the subpopulation of recruited MDSCs varies depending on the organ. In this respect, the PMN-MDSCs phenotype was predominant over the Mo-MDSCs phenotype in PP and spleen, while a balance between both subpopulations was detected in MLN. Our results, based on MDSCs depletion by treatment with GEM, showed that splenocyte proliferative activity in infected mice was restored after polyclonal and antigen-specific stimulation and accompanied by a significant reduction in NO levels. Gemcitabine (GEM) is an antimetabolite widely used to deplete MDSC (Le et al., 2009; Chesney et al., 2017). Although it has been reported that it can also affect all those cells that proliferate rapidly, the administration of low doses of GEM does not affect the number of CD4+ T cells, CD8+ T cells, B cells or macrophages (Suzuki et al., 2005). These findings indicate that, during Ye infection, MDSCs migrate to the spleen and suppress the immune response through a NO-dependent mechanism. Several studies have demonstrated that MDSCs induce immunosuppression in the host response to infection and some of them suggest that NO production by these cells is responsible for T-cell suppression (Cuervo et al., 2011; Ribechini et al., 2019). On the other hand, after the depletion of MDSCs, the proliferative activity of MLN cells remains unaffected upon polyclonal stimulation. However, an increase in proliferative activity was observed when cells were stimulated with Ye-specific antigens. These findings suggest that MDSCs that are expandedduring Ye infection and accumulate in the intestinal mucosa may regulate the immune response in an antigen-specific manner. Meanwhile, those that accumulate in the spleen may regulate the immune response in a non-specific way. The significance of this discovery may not be immediately evident, but it could represent a component of the protective mechanisms triggered to counter the excessive tissue damage or p.i. complications that can occur as a result of an exacerbated immune response. In this context, MDSCs have been reported to be critical regulators of inflammation during *Trypanosoma cruzi* infection (Arocena et al., 2014). Also, MDSCs play a crucial role in regulating inflammation as demonstrated in acute bacterial pneumonia caused by *Klebsiella pneumoniae* and in cystic fibrosis patients with chronic *Pseudomona aeruginosa* infection (Poe et al., 2013; Rieber et al., 2013). Furthermore, Ye is a bacterium that is linked to the onset of reactive arthritis, a condition in which the immune response is dysregulated (Colmegna et al., 2004; Eliçabe et al., 2010; Silva et al., 2020; Zeidler and Hudson, 2021). Based on this background, we hypothesize that MDSCs may regulate the antibacterial response in order to limit inflammation and prevent post-infectious sequelae related to an exacerbation of the immune response.

The expansion of MDSCs can be triggered by both Gram-positive and Gram-negative bacteria. It has been proposed that PMN-MDSCs mainly expand in infections caused by Gram-positive bacteria, while Mo-MDSCs are induced regardless of the Gram-staining (Medina and Hartl, 2018). According to our research, Ye induces the expansion of PMN-MDSCs and Mo-MDSCs during infection. However, only Mo-MDSCs have the ability to suppress T-cells. We observed that the function of T-cells was restored when we inhibited the iNOS enzyme, indicating that Mo-MDSCs exert their suppressive activity through a mechanism that depends on iNOS enzyme activity and NO production. Interestingly, the co-culture of T-cells with MDSCs-depleted splenocytes did not modify their proliferative capacity, suggesting that Mo-MDSCs may be the cells with the highest suppressive activity during the infection with Ye. The expansion of Mo-MDSCs was reported in many infectious diseases caused by Gram-negative bacteria (Dorhoi and Du Plessis, 2018). In fact, it has been demonstrated that LPS, the major cell wall component of Gram-negative bacteria, induces the differentiation of Mo-MDSCs (Poe et al., 2013). As previously stated, Mo-MDSCs have versatile roles in an infection, with either beneficial or detrimental outcomes for the host depending on the pathogen and the infection. According to the study by Tam et al., moderate amounts of Mo-MDSCs can protect the host against immune-mediated pathology during *Salmonella typhimurium* infection. However, if the accumulation of these cells exceeds a certain threshold, it may lead to immunosuppression and can prolong the infection (Tam et al., 2014). Our research has shown that mice infected with Ye can successfully overcome the infection. In fact, no bacteria were detected in the spleen or intestinal mucosa after three weeks despite the presence of MDSCs in these organs. This indicates that Mo-MDSCs do not hinder the anti-bacterial response and do not promote bacterial persistence. This finding supports the hypothesis that Mo-MDSCs may provide protection against immune-mediated pathologies associated with an infection.

In conclusion, our results highlight a mechanism by which Ye may induce suppression of the immune responses. We suggest that, during Ye-infection, NO-producing Mo-MDSCs expand and accumulate in MLN and spleen. These cells can then suppress T-cell function without interfering with the anti-bacterial effector response. Instead, these immature myeloid cells could play an important role in regulating the inflammatory response and protecting affected tissues.

## Data Availability

The original contributions presented in the study are included in the article. Further inquiries can be directed to the corresponding author.
